# Long-term behaviour of non-functioning pituitary microadenomas: experience from a tertiary care centre in Romania

**DOI:** 10.3389/fendo.2025.1613239

**Published:** 2025-08-11

**Authors:** Mădălina Elena Iftimie, Iulia Florentina Burcea, Ramona Dobre, Stella Pigni, Flavia Prodam, Cătălina Poiană

**Affiliations:** ^1^ Department of Endocrinology I,’’C.I.Parhon” National Institute of Endocrinology, Bucharest, Romania; ^2^ Department of Endocrinology, “Carol Davila” University of Medicine and Pharmacy, Bucharest, Romania; ^3^ Department of Health Sciences, University of Piemonte Orientale, Novara, Italy

**Keywords:** pituitary, nonfunctioning microadenoma, hypopituitarism, tumor growth, surveillance

## Abstract

**Objective:**

An optimal surveillance plan of micro-nonfunctioning pituitary adenomas (micro-NFPAs) is not well established despite high prevalence and increasing incidence of these tumors. This study aims to characterize the natural history of conservatively treated micro-NFPAs and provide evidence for a management algorithm.

**Methods:**

Retrospective, single center cohort study that analyzed clinical, hormonal and imaging data of conservatively managed micro-NFPAs (years 2018-2023).

**Results:**

371 patients with micro-NFPAs were included in the study (mean age at diagnosis 41.26 ± 13.71 years, 91.6% females) with a mean tumor size at detection of 5.51 ± 1.95 mm. Over a median follow-up period of 4.8 years (IQR 2-8.64): 23.7% of all micro-NFPAs were stable, 41% regressed and 35.3% had any progression in size (34.5% of patients had a significant tumor growth, when considering 1 mm enlargement as significant, with a growth incidence of 17.18 per 100 person-years, 95% CI: 14.2- 20,15). The median growth was 1 mm (IQR: 0.5-2) over the entire follow up period and only 2.42% microadenomas evolved into macroadenomas, without clinical consequences. Sex, BMI, age were not predictors of tumor growth, however tumors smaller than 6 mm had a 47.4% higher incidence rate of significant tumor growth (≥ 1mm) events per 100 person-years, compared to larger microadenomas. Alternating CT with MRI during follow-up is an important predictor for tumor variability. Median time until growth was 11.32 months (95%CI: 9.66- 12.97). At diagnosis, 1.1% had secondary hypogonadism, 1.1% hypothyroidism and 0.5% secondary hypoadrenalism. During follow-up, only 5 patients (1.3%) developed hypopituitarism after a median of 2 years (0.9-5.1), irrespective of tumor enlargement or other demographic and clinical factors.

**Conclusion:**

Micro-NFPAs have an overall benign clinical course, with a high measuring variability in tumors smaller than 6 mm and hypopituitarism is a very rare occurrence. Performing the first follow-up MRI at one year and if stable, delaying re-evaluation to 5 years, without pituitary function reassessment in absence of clinical manifestations, is a safe and cost-effective approach.

## 1 Introduction

Non-functioning pituitary adenomas (NFPAs) are benign tumors of the pituitary gland without signs of hormone hypersecretion (1). They constitute about one-third of all pituitary adenomas and are the second most frequent pituitary lesion after prolactinoma (1, 2). According to their size, by convention, they are classified in microadenomas (maximum diameter < 1 cm) and macroadenomas (at least 1 cm in size) (3). While the latter usually presents with compression symptoms of nearby structures, non-functioning pituitary microadenomas (micro-NFPAs), given their smaller dimension, are frequently, an incidental discovery (3). The incidence of micro-NFPAs varies from 10.4% to 14.4% in autopsy studies performed in patients who died from pituitary-unrelated causes to 10-38% in radiological examinations (4–7). The incidence has increased considerably over the last decades, mainly due to increases in the availability, use and resolution of magnetic resonance imaging in developed countries (8). Despite this rising trend, optimal management strategies for micro-NFPAs are not established, existing guidelines comprising important differences in practice, between countries (9–11). At the root of this uncertainty lie significant gaps in the knowledge regarding the long-term outcome of these tumors, probably due to their prolonged natural history, and their variability in clinical behaviour. The management of non-functioning pituitary tumors relies on the risk of tumor growth and new pituitary deficiencies. Studies to date have reported micro-NFPA associated growth and hypopituitarism rates that vary between 0-52.6% and 0-50%, respectively (12–22). Lack of evidence-based recommendations for the length or frequency of biochemical and radiological surveillance can lead to significant psychological burden for the patient and to considerable financial costs for the healthcare provider. Moreover, recent data on MRI monitoring of pituitary tumors showed an accumulation of gadolinium in brain tissue, so reducing unnecessary scans is advised (23).

We conducted a retrospective cohort study that aims to evaluate the natural history of conservatively treated non-functioning pituitary microadenomas and assess tumor behaviour over long-term follow-up, with a focus on the potential for tumor enlargement and/or pituitary hormone deficiencies occurrence.

## 2 Patients and methods

### 2.1 Study design and participants

We conducted a retrospective, cohort study of patients with micro-NFPAs who received care at the “C. I. Parhon’’ National Institute of Endocrinology, Department of Endocrinology I, between January 1^st^ 2018 and 31^st^ December 2023. We performed a non-interventional review of medical charts and retrieved clinical, hormonal and pituitary imaging data (computed tomography scans and magnetic resonance imaging 1.5 T with pituitary protocol) at diagnosis and at each evaluation during follow-up. We selected patients with pituitary masses suggestive of adenomas on imaging, smaller than 1 cm in size and without clinically and biochemically proven hormone hypersecretion. Only patients with at least 2 separate evaluations in our clinic were included in the study. The frequency of hormonal and imaging evaluations was established by each physician, according to their preference/current European guidelines/clinical picture of the patient.

### 2.2 Pituitary function evaluation

Pituitary hormone deficiencies (PHD) were defined as follows: secondary hypogonadism as low or inappropriately normal follicle-stimulating hormone (FSH) and luteinizing hormone (LH) paired with low morning testosterone in males or low oestradiol and oligo/amenorrhea in women of reproductive age and, in post-menopausal women FSH and LH below the age reference range was the only criterion; secondary hypothyroidism was defined as low or inappropriately normal thyroid stimulating hormone (TSH) coupled with low free-thyroxine, secondary hypoadrenalism was confirmed by dynamic testing (short cosyntropin stimulation test or insulin tolerance test) and the need of substitution therapy with prednisone/hydrocortisone. Growth hormone deficiency (GHD) was defined by low values of insulin-like growth factor 1 (IGF1) in at least two occasions. Patients with PHD were carefully screened for other causes of hypopituitarism such as concomitant medication.

### 2.3 Evaluation of tumor growth

Tumor size evolution was assessed with serial imaging (CT or MRI scans with iodine and gadolinium-based contrast, respectively) both in our clinic and other institutions. All MRI scans taken into consideration used a magnetic field strength of 1.5 Tesla and a protocol for pituitary gland evaluation. Pituitary adenomas were categorized as having progression or regression if any increase or decrease in size was recorded. Significant progression was defined as an increase in any diameter with a minimum of 1 mm. Micro- NFPAs were classified as stable if they remained unchanged in size. Tumor size at diagnosis was evaluated both as a continuous and a dichotomous variable (< 6 mm, ≥ 6 mm).

### 2.4 Vision assessment

Visual deficit was appraised by visual field testing in patients with subtle clinical complaints or signs.

### 2.5 Statistical analysis

All statistical tests were performed using IBM SPSS Statistics for Windows, Version 30.0 (IBM Corp., Armonk, NY, USA). Continuous variables were expressed as means with standard deviations or medians with interquartile range (IQR) and percentages were used to illustrate categorical variables. For the analysis of the latter Chi-squared tests were performed. Independent samples T test and Mann-Whitney non-parametric test was used for the comparison of continuous or ordinal variables. A logistic regression analysis was conducted to examine the effects of various predictors on the likelihood of tumor growth or pituitary insufficiency among patients. Kaplan-Meyer survival curves were generated with the event of interest being tumor growth. Patients who did not experienced tumor growth by the last evaluation were censored. The log-rank test was applied to assess differences in survival curves between subgroups.

## 3 Results

### 3.1 Patient and tumor characteristics

Out of the 371 patients included in the study, 340 were females (91.6%) and only 31 were males (8.4%). The mean age at diagnosis was 41.26 ± 13.71 years and on the initial imaging, the mean tumor size was 5.51 ± 1.95 mm, with no difference between sexes (**Table 1**). Initial imaging modality was CT in 57.7% cases and MRI in 42.3% cases. More than half of the micro-NFPAs included had a maximum tumor diameter at baseline smaller than 6 mm, while 39.9% of tumors were ≥ 6 mm. Only 5.4% of all tumors included had a cystic component.

**Table 1:** Patient demographics and tumor characteristics.

Variable	Micro-NFPA (n = 371)
Number of females (n,%)	340, 91.6%
Mean	
Age at diagnosis (years)	**41.26 ± 13.71**
Age at diagnosis (years), females	41.32 ± 13.78
Age at diagnosis (years), males	40.64 ± 13.00
Body mass index (kg/m²)	**27.16 ± 6.28**
Body mass index (kg/m²), females	27.02 ± 6.35
Body mass index (kg/m²), males	28.68 ± 5.36
Tumor diameter at detection (mm)	**5.51 ± 1.95**
Tumor diameter at detection (mm), females	5.53 ± 1.96
Tumor diameter at detection (mm), males	5.27 ± 1.85
Micro-NFPAs with initial maximum diameter < 6 mm (n,%)	**223, 60.1%**
Micro-NFPAs with initial maximum diameter ≥ 6 mm (n,%)	**148, 39.9%**
Micro-NFPAs with cystic component (n,%)	**20, 5.4%**
Presence of double micro-NFPAs on initial imaging (n,%)	**63, 17%**

Values in bold represent data for the total study population; non-bolded values are stratified by sex.

### 3.2 Tumor size during follow-up

**Figure 1:**
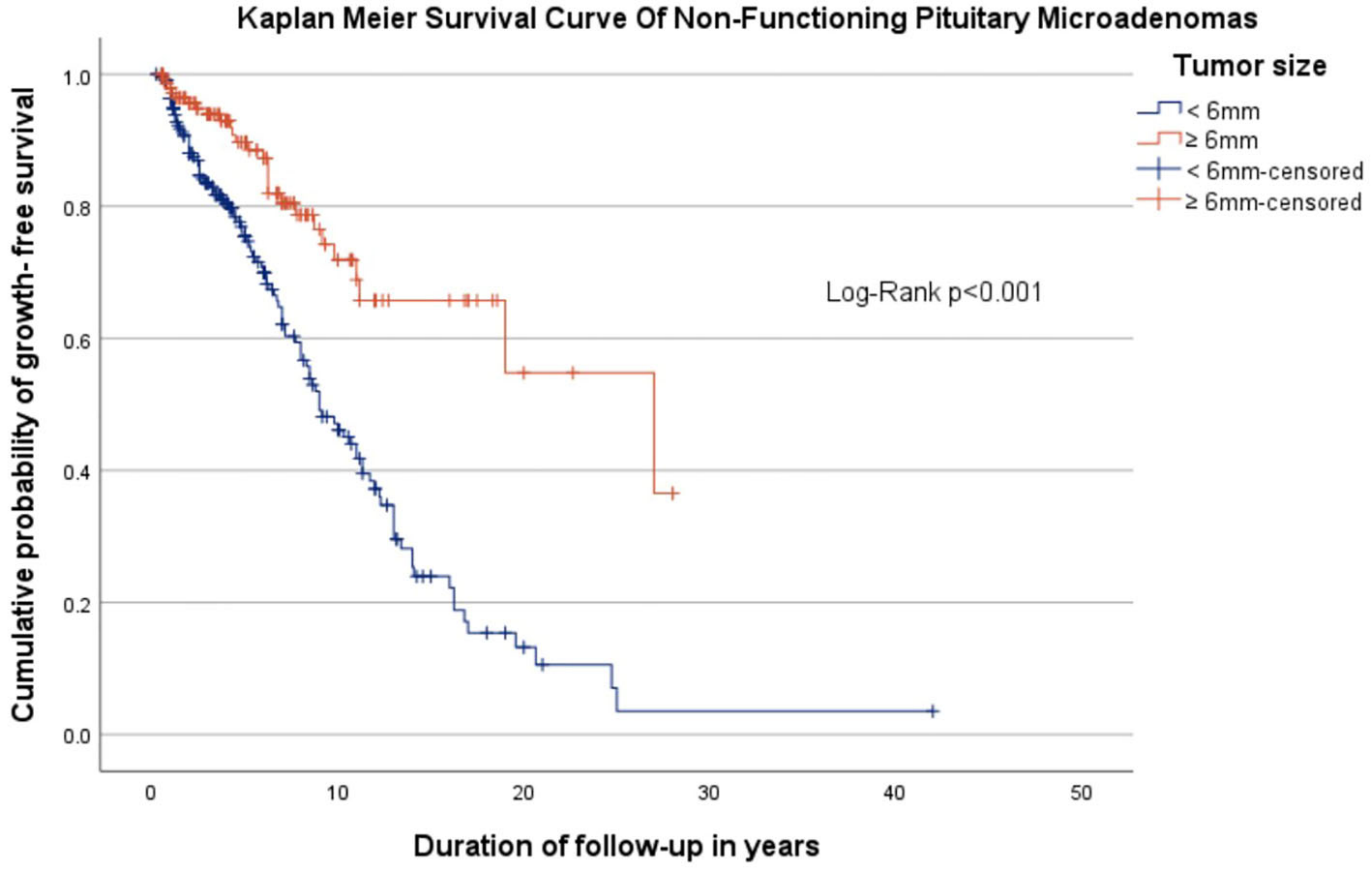
Kaplan Meyer survival curve showing the cumulative probability of growth-free survival in patients with non-functioning pituitary microadenomas, stratified by tumor size, over follow-up period.

All 371 patients were followed-up with different imaging modalities (CT and MRI in accordance to method availability, contraindications and patient preferences) over a median period of 4.8 years (IQR 2-8.64). A proportion of 75.2% patients were followed with the same imaging modality used at diagnosis, while 24.8% patients, initially assessed with CT or MRI, were reevaluated with alternating CT or MRI scans. The patients received a median of 3 scans (interquartile range 2-4) during this interval. 23.7% of all micro-NFPAs analyzed were stable, 41% regressed and 35.3% had a progression in size, with differences between baseline tumor-size subgroups. Tumors smaller than 6 mm at diagnosis had a higher progression rate (46.6%) versus larger tumors (18.2%), while the latter had a significantly higher rate of regression compared to the former (63.5% vs. 26%, **Table 2**). Tumors who progressed had a median increase in size of 1 mm (IQR: 0.5-2) over the entire follow up period, regardless of the initial tumor size (**Table 3**). Only 9 (2.42%) microadenomas evolved into macroadenomas at last follow-up (mean size = 11.1± 2.96 mm). The maximum diameter at diagnosis also influenced the time until tumor growth. The median period of time until tumor growth, for tumors smaller than 6 mm was 9 months (95% CI, 7.27 - 10.73), whereas for tumors 6 mm or larger, the median time to tumor growth was 27 months (95% CI, 9.22 - 44.78). The difference was statistically significant (log rank p< 0.001, **Figure 1**). Sex and age at diagnosis did not influence time until tumor growth on Kaplan-Meier survival analysis or Cox Regression model. Overall, when assessing the evolution of all micro-NFPAs included in our cohort, the average yearly growth rate was null (median of 0.0 mm, IQR: -0.31 – 0.1 mm).

**Table 2:** Imaging outcomes across initial tumor-size groups.

Tumor	Non-functioning pituitary microadenomas			P value
	All (n = 371)	< 6 mm (n = 223)	≥ 6 mm (n = 148)	
Progression (n,%)	131, 35.3%	104, 46.6%	27, 18.2%	**< 0.001**
Stable (n, %)	88, 23.7%	61, 27.4%	27, 18.2%	0.043^*^
Regression (n, %)	152, 41%	58, 26%	94, 63.5%	**< 0.001**
Incidentally discovered (n,%)	112, 30.2%	58, 26%	54, 36.4%	

*After applying the Bonferroni correction for multiple comparisons (α adjusted=0.0167), this p-value is not statistically significant. Bolded p-values indicate statistically significant differences (p < 0.05).

**Table 3:** Pattern of tumor progression.

Median	Micro-NFPAs who progressed in size during follow-up			P value
	All (n=131)	< 6 mm (n = 104)	≥ 6 mm (n = 27)	
Increase in size, mm (IQR)	1 (0.5 - 2)	1 (0.5-2)	1 (0.6-2)	0.862
Time until growth, months (95% CI)	11.32 (9.66 -12.97)	9 (7.27 - 10.73)	27 (9.22 - 44.78)	**< 0.001**

Bolded p-values indicate statistically significant differences (p < 0.05).

If we consider an increase in tumor size of minimum 1 mm as being significant, then 34.5% of patients had a significant tumor growth, with a growth incidence of 17.18 per 100 person-years (95% CI: 14.2- 20,15). Patients with tumors less than 6 mm had a 47.4% higher incidence rate of significant tumor growth events per 100 person-years, compared to patients with tumors 6 mm or larger (incidence rate ratio = 1.474, 95% CI (1.014, 2.142), p = 0.042).

A logistic regression analysis was conducted to assess the effects of various predictors on the likelihood of tumor growth among patients (**Table 4**). Maximum tumor diameter at diagnosis was a significant predictor of tumor growth. Patients with tumors less than 6 mm had higher odds of experiencing tumor growth compared to those with tumors 6 mm or larger (OR - 4.89, P<0.001, 95% CI: 2.82-8.49). The modality of follow-up and the number of scans performed, significantly influenced tumor growth. Notably, patients who were rarely followed and underwent a higher number of scans had significantly higher odds of tumor growth. The choice of initial imaging (MRI vs CT) or the consistency in imaging modality use across monitoring did not predict tumor growth.

**Table 4:** Logistic regression predicting tumor growth.

Predictor variable	OR	95% CI	P value
Age at diagnosis	0.99	0.98-1.01	0.798
BMI	0.99	0.96-1.04	0.940
Sex, female	1	0.42-2.39	0.994
Smoker status	1.05	0.72-1.55	0.783
Incidental tumor discovery	0.95	0.54-1.67	0.862
Tumor with cystic component	0.7	0.24-2.06	0.522
Presence of double microadenomas	1.78	0.9-3.49	0.097
Pituitary insufficiency at diagnosis	0.31	0.08-1.22	0.093
Tumor size < 6 mm,at diagnosis	4.68	2.74-7.99	<**0.001**
Total follow-up period	0.97	0.91-1.04	0.416
Initial imaging modality (MRI vs CT)	1.41	0.76-2.61	0.271
Imaging consistency	0.75	0.42-1.32	0.321
Number of scans	1.44	1.15-1.79	**<0.001**
Follow-up modality^*^	1.42	1.15-1.75	**<0.001**

OR, odds ratio; CI, confidence interval; *biannual, yearly, at 2, 3, 4, 5 or more years. Bolded p-values indicate statistically significant differences (p < 0.05).

Another logistic regression analysis model was generated in order to assess the influence of imaging modalities on tumor size variability during follow up (**Table 5**). The use of the same imaging technique across follow-up decreased the proportion of tumor size variability (growth and reduction in size) by almost 80% (p=0.018). A higher number of scans, irrespective of monitoring duration and the presence of double pituitary microadenomas were linked to a higher rate of tumor growth or regression (OR = 1.882 and 3.197, respectively, p<0.001). However, this model explained approximately 24% of the tumor variability, with excellent sensitivity (95.4%) but limited specificity (27.3%).

**Table 5:** Logistic regression predicting tumor size variability across follow-up.

Predictor variable	OR	95% CI	P value
Age at diagnosis	0.99	0.98-1.02	0.659
Sex,female	0.49	0.2-1.22	0.128
Smoker status	0.82	0.53-1.26	0.368
Tumor with cystic component	1.26	0.39—4.05	0.697
Presence of double microadenomas	3.2	1.62-6.3	**<0.001**
Tumor size < 6 mm, at diagnosis	0.59	0.34-1.05	0.076
Total follow-up period	0.95	0.88-1.03	0.227
Initial imaging modality (MRI vs CT)	0.75	0.16-3.55	0.721
Imaging consistency	0.205	0.05-0.76	**0.018**
Interaction between initial and follow up imaging modalities	1.38	0.26-7.22	0.700
Number of scans	1.88	1.4-2.53	**<0.001**
Follow-up modality^*^	1.256	0.99-1.6	0.066

OR, odds ratio; CI, confidence interval; *biannual, yearly, at 2, 3, 4, 5 or more years. Bolded p-values indicate statistically significant differences (p < 0.05).

Follow-up frequency (**Table 6**) was not influenced by demographic factors, tumor size or growth pattern. Patients with cystic tumors had a slight tendency to be more frequently monitored (r = 0.118, p = 0.023).

**Table 6:** Frequency of micro- NFPAs follow-up.

Follow-up modality	All (n= 371)	< 6 mm (n=223)	≥ 6 mm (n=148)	P value
Biannual (n,%)	29, 7.8%	11, 4.9%	18, 12.2%	0.178
Yearly (n,%)	164, 44.2%	103, 46.2%	61, 41.2%	
Biennial (n,%)	103, 27.8%	67, 30%	36, 24.3%	
At 3 years (n,%)	34, 9.2%	21, 9.4%	13, 8.8%	
At 4 years (n,%)	18, 4.9%	9, 4%	9, 6.1%	
At ≥ 5 years (n,%)	23, 6.2%	12, 5.4%	11, 7.4%	

Tumor apoplexy was not observed in this cohort at any moment during monitoring.

### 3.3 Pituitary function

10 patients (2.7%) had pituitary hormone deficiency (PHD): 4 patients were reported to have secondary hypogonadism (1.1%, one had associated arginine vasopressin deficiency, AVP-D), 4 had secondary hypothyroidism (1.1%), and 2 patients had secondary hypoadrenalism (0.5%). Tumor size at baseline was not an influencing factor of pituitary insufficiency: half of patients had microadenomas < 6 mm and the other half had ≥ 6 mm, although patients with PHD had slightly larger tumors (6.16 ± 1.9 mm versus 5.49 ± 1.95 mm, p=0.288). Age, sex, smoker status, BMI, presence of double microadenoma or cystic component did not correlate with hypopituitarism. All patients underwent repeat assessment of pituitary function during follow-up and new PHD was detected in 5 patients (1.3%) after a median of 2 years (0.9-5.1). 3 of them were reported to have secondary hypogonadism (1 recovered after 6 months), one transitory growth hormone deficiency and the other one secondary hypothyroidism. None of them had PHD at baseline. There were no statistically significant predictors for developing a new pituitary insufficiency, although female sex might have a slightly higher propensity for developing PHD (OR – 3.55, 95% CI: 0.35-35.5, p=0.281).

Pituitary function surveillance frequency was similar in patients with or without PHD, but had a longer duration in patients with tumors ≥ 6 mm (median 6.28 years, 3.58-9.06) compared with patients with smaller tumors, <6 mm (median 5 years, 2-8.64, p= 0.032).

### 3.4 Visual assessment

Visual deficits were present in only two patients (one male and one female) with tumor sizes at diagnosis of 2 mm and 7.5 mm, respectively. Only the former patient had tumor growth (last known tumor diameter- 4 mm). No patient required surgery.

### 3.5 Multiple endocrine neoplasia type 1 association

Of all patients analyzed, 5 had multiple endocrine neoplasia type 1 syndrome (2 females and 3 males) with a mean age of 40.4 ± 14.7 years. Micro-NFPAs in these cases were discovered after pituitary imaging performed for screening purposes and had a mean diameter of 6.36 ± 2.06 cm. None of them had tumor growth or pituitary insufficiency, either at diagnosis or during follow-up.

### 3.6 Double micro-NFPAs

17% of patients analyzed had double micro-NFPAs on imaging (82.5% were metachronous with the second microadenoma being discovered after a mean of 57.15 months). Tumor growth and regression rates were similar to single micro-NFPAs (26.9% vs 37%, p=0.129 and 36.5% vs 41.8%, p=0.429). Stable tumors appear significantly more frequent in double adenomas versus single lesions (36.5% vs 21.1%, p= 0.009), but when correcting for other confounding factors in multivariate analysis, double micro-NFPAs are in fact a predictor for tumor size variability (**Table 5**). 2 patients with double micro-adenomas presented hypopituitarism at initial evaluation and only one during follow-up, with no significant differences compared to single adenomas. Only one patient associated double- micro NFPAs with MEN 1 syndrome.

## 4 Discussion

According to our knowledge, this is the second largest study to date, following a multi-center study conducted in 23 UK endocrine departments (21) and the largest single-center cohort study to evaluate the natural history of micro-NFPAs conservatively managed.

In our study, we found that 35.3% of microadenomas had any increase in size and a similar percent, 34.5% grew with at least 1 mm compared with the initial evaluation. These results are consistent with another study from Sam et al. (18) who also reports a high percentage of tumor growth over a similar mean follow-up period. In comparison, other studies show a lower probability of an increase in size in microadenomas, that ranges between 7.4% and 13.5% (12, 13, 16, 17, 20–22, 24). The high tumor size variability (76.3% either progressed or regressed) noted in this study is partially explained by the inconsistency in the use of imaging modalities across monitoring (CT alternating with MRI), the number of scans performed and the long surveillance. We observed a growth incidence of 17.18 per 100 person-years, which is higher than previously reported (13, 21, 25–27), possibly due to a longer follow-up duration, with a median of 4.8 years.

Opposite to other existing studies, we found that tumor diameter at diagnosis was a significant predictor of growth (13, 17, 21, 22). Tumor growth rate was different between the two subgroups analyzed: tumors smaller than 6 mm had an approximately 4.9 chance of growth compared with tumors larger than 6 mm, with a median of 9 months until growth versus 27 months. Considering that inconsistency in the imaging method performed (CT alternated with RMN scans in our cohort on account of availability, patient’s preference, contraindications at certain time points and costs) was not a predictor for tumor growth in this subgroup of patients, these results may be justified by other several limitations: interobserver variability and possibly a higher error of measuring microadenomas smaller than 6 mm. It is also important to consider several pitfalls of neuroimaging that can be encountered and confound the radiological assessment of microadenomas, such as technical artifacts (image noise due to different signal intensities of adenohypophysis, bony structures, sinus air content or carotid arteries), pituitary hyperplasia and variants of normal anatomy (thicker bony components, normal hypersignal of neurohypophysis) (28, 29).

Other significant factors that were found to influence tumor growth in our study were the modality of follow-up and the number of scans performed. More specifically, patients who underwent a higher number of scans with longer periods of time between scans, were more likely to experience microadenoma growth.

Acknowledging the fact that reader discrepancies in radiological studies vary between 25%-40% and the same lesion can demonstrate changes in its diameter, even upon immediate re-imaging (due to patient’s positioning, breathing, swallowing), measurement variability, especially in lesions smaller than 6 mm, along with the above mentioned particularities and the long observation period, can account for the high rate of tumor growth observed in our study (30). No relationship between sex, age and tumor enlargement was observed in our study, consistent with previous observations (13, 21, 22), although microadenomas were more frequently present in women compared to men, with a pooled micro-NFPAs incidence of 72.46% in females in other studies (12–14, 16, 27, 31, 32). This difference in micro-NFPAs prevalence between sexes following radiological studies, despite similar prevalence being observed in autopsy studies (33), can be an effect of the lower medical addressability and delays in diagnostic in the male population. Di Somma et all (34) hypothesized that this skewed distribution between sexes may be influenced by sex hormone profiles. The gender imbalance seen in this study may impact the generalizability of the results in the male population.

Another interesting aspect identified by the present study was the prediction value of double micro-NFPAs for tumor dimension variability (progression and regression), when adjusting for other confounding factors such as age, gender, BMI, tumor size at baseline, imaging modalities and monitoring duration. Double micro-adenomas are a rare occurrence, with a reported prevalence of 0.9% in autopsy studies (35) and data regarding their natural history are sparse, consisting mainly of case reports of functional double micro-adenomas (36).

When comparing tumor size variability with other observational studies performed on Romanian population, although scarce, we notice similar progression and regression rates of non-functioning pituitary microadenomas, as reported by Carsote et al. (37). They analysed patients referred to the same tertiary center, only several years prior to our study. This suggests that despite an evolving surveillance approach and a broader use of MRI technology, the natural history of pituitary microadenomas remained unchanged and slightly different compared to existing data from other countries. This could be explained by the Romanian health care system particularities, where patients can access medical care in tertiary centres, regardless of pathology type or severity, thus excluding selection bias and having a good representation of nationwide populations. Another possible incriminating factor could be represented by a distinct genetic profile of Romanian population. Data regarding genetic testing is scarce in microadenoma cohorts in our country, only one study showing that there is a p.R16H AIP sequence variant that is relatively frequent in Romanian sporadic pituitary adenoma patients (38). Genetic testing could not be performed in the present study due to its retrospective, observational nature, but undergoing genetic, as well as pituitary tumor microenvironment analyses could better characterize micro -NFPAs behaviour and help individualize surveillance (39).

Given the paucity of long-term data regarding the natural history of micro-NFPAs, the follow-up strategy in terms of length or frequency is not well established. Current recommendations rely heavily on clinical experience and show slight discrepancies between countries. The Endocrine Society guidelines recommend MRI reevaluation of microadenomas 1 year after detection and if stable, at 1–2 years for the next 3 years and less often thereafter (9). Comparably, the German guidelines on therapy of clinically non-functioning pituitary tumors recommend yearly MRI scans for the first 3 years, with further controls according to individual assessment (11). On the other hand, some experts do not recommend imaging surveillance for microadenomas smaller than 5 mm (28, 40) and for those lesions > 5 mm, morphologic surveillance in 6-month MRI is recommended and if there is no progression, MRI can be repeated at 2 years and then stopped (40). Two of the largest cohort studies, apart from the present study, suggest that delaying the first follow-up RMN to 3 years after the initial evaluation is a safe approach (13, 21). Furthermore, Galland et al. observed that fewer than 5% of microadenomas grow to more than 1 cm over long-term follow-up, without relevant consequences (40). Considering these observations and that in our study the median time until growth for all lesions was approximately 11 and a half months and the absolute change in size over the median follow-up period of almost 5 years was on average only 1 mm, with only 2.42% exceeding 10 mm, without developing clinically relevant signs and symptoms or requiring surgery, a radiological follow-up at 1 year after detection, and if stable at 5 years is a safe and cost-effective surveillance strategy.

When assessing pituitary function after microadenoma detection, only a minority had pituitary hormone deficits at initial clinical visit – 2.7%, of whom 1.1% had secondary hypogonadism, 1.1% central hypothyroidism and 0.5% secondary hypoadrenalism. Our data estimate that hypopituitarism at baseline is a very rare occurrence compared with previous studies that show a prevalence between 5.3% and 11.1% (12, 13, 21, 27). At opposite poles are two studies that found a prevalence of pituitary hormone deficits of 0% (16, 22) and another 3 studies that reported PHD in 33.3%-50% cases (14, 15, 18). These discrepant findings could be partly explained by the small sample size (up to 38 microadenomas, only one study included 90 patients), but they are most likely caused by significant heterogeneity in patient selection, use of diagnostic tests and criteria for defining hypopituitarism. Underlying pathological conditions may influence these results, for example in one of the aforementioned studies, who discovered an impaired GH response to GHRH-arginine stimulation test in 50% of patients, the mean BMI was 36.2 kg/m2, significantly higher than those who had a normal GH response or healthy controls (15). In our study, the mean BMI was only slightly elevated (27.16 ± 6.28 kg/m²) and was not correlated with hypopituitarism occurrence.

Similarly to other large cohort studies, in this study, hypogonadism and hypothyroidism were the most common pituitary deficiencies (13, 21), while other studies report growth hormone deficiencies (15) or hypoadrenalism as more frequently associated with micro-NFPAs (12, 14).

Interestingly, although patients with hypopituitarism had a slightly larger tumor diameter than those with normal pituitary function (6.16 ± 1.9 mm versus 5.49 ± 1.95 mm), 50% of patients with PHD had a diameter smaller than 6 mm. Therefore, the maximum tumor diameter in microadenomas does not influence the presence of hypopituitarism, as highlighted by other studies (12, 21). This observation challenges the opinions of some experts that do not recommend pituitary insufficiency evaluation at diagnosis in microadenomas smaller than 6 mm (9, 11). On the same note, the Endocrine Society and French Endocrinology Society do not recommend hormonal surveillance for microadenomas whose clinical or radiological picture does not chance over time (9, 10, 40). In our cohort, all patients were reevaluated for pituitary hormone deficits, regardless of known tumor growth. The same practice is adopted by 47.2% of UK clinicians who responded to an online survey performed in 2021-2022, would reassess pituitary function annually until discharge, in absence of tumour growth or new relevant symptoms, especially those working in tertiary care hospitals (41). In concordance with other series (13, 21, 27), a very small percentage of patients (n=5,1.3%) developed pituitary insufficiency, after a median of 2 years: 3 had secondary hypogonadism, 1 transient GHD and the other secondary hypothyroidism. We could not identify any predictor of new onset PHD. Tumor growth, sex, age, pituitary insufficiency at baseline did not influence the development of new hypopituitarism during follow-up. Although statistically non-significant, female sex showed a propensity for developing new PHD, possibly explained by the greater amount of females included in our study. Our results suggest that there is little to no value in reassessing pituitary function in microadenomas who do not develop new relevant clinical symptoms or signs.

In multiple endocrine neoplasia type 1 syndrome, pituitary tumors seem to be less aggressive than previously suggested. A study that evaluated the natural course of microadenomas in a large MEN1 cohort showed that only 9.7% had minimal tumor growth over a follow up period of 5.3 years, none progressing to a macroadenoma. Similarly, tumor growth or pituitary deficiency was not detected in MEN 1 patients included in our cohort (42).

The present study has several strengths: the large number of patients included (to the best of our knowledge this is the largest single-centre retrospective cohort study to date), the long follow-up duration and the homogenous evaluation of pituitary function. Furthermore, patients with pituitary hormone deficits were carefully screened for other possible confounding factors and those with other causes of PHD were not included in the analysis. Amongst study limitations we can identify those inherent to its retrospective nature, the use of both CT and MRI in evaluating pituitary gland lesions, possible variations in imaging interpretation, considering that some imaging studies were performed in other institutions. We also acknowledge the possibility that some microadenomas, especially those smaller than 6 mm may be in fact, technical artifacts, variants of normal anatomy or pituitary hyperplasia, which can explain the greater variability in size compared to microadenomas > 6 mm. Due to its observational, non-interventional nature, the present study cannot further explore molecular and immunohistochemical features that could explain tumor behaviour and female preponderance in our cohort. Other prospective or experimental studies are required to validate our observations.

In conclusion, this present large retrospective cohort study emphasises the benign natural course of non-functioning pituitary microadenomas over a long follow-up period (median 4.8 years). Even though almost 35% of microadenomas displayed growth, the absolute change in size over the whole surveillance timeframe was 1 mm, only 2.42% progressed to macroadenomas, without developing relevant clinical signs or symptoms. Considering that time until growth was approximately 11.3 months, performing the first follow-up MRI at one year and delaying re-evaluation to 5 years if stable is a safe and cost-effective approach that reassures both clinicians and patients. We advise against pituitary function reassessment in patients without pituitary insufficiency at baseline, given the very low risk of developing new PHD over time and the lack of predictors. We advocate for the use of MRI as the preferred method of radiological surveillance for microadenomas and encourage physicians to be aware of the possible technical artifacts that may influence the image interpretation. Consistency in the use of imaging modalities during radiological monitoring of micro-NFPAs is a crucial aspect for an accurate evaluation of tumor growth or shrinkage. If during follow-up, the patient exhibits symptoms suggestive of mass effects or pituitary insufficiency, prompt re-evaluation should become a priority.

## Data Availability

The raw data supporting the conclusions of this article will be made available by the authors, without undue reservation.
